# Long-wavelength macromolecular crystallography – First successful native SAD experiment close to the sulfur edge

**DOI:** 10.1016/j.nimb.2016.12.005

**Published:** 2017-11-15

**Authors:** O. Aurelius, R. Duman, K. El Omari, V. Mykhaylyk, A. Wagner

**Affiliations:** a Diamond Light Source, Harwell Science and Innovation Campus, Didcot OX11 0DE, United Kingdom; b Research Complex at Harwell, Harwell Science and Innovation Campus, Didcot OX11 0FA, United Kingdom

**Keywords:** Macromolecular crystallography, X-ray diffraction, Sulfur SAD, Soft X-rays, Crystallographic phase problem

## Abstract

Phasing of novel macromolecular crystal structures has been challenging since the start of structural biology. Making use of anomalous diffraction of natively present elements, such as sulfur and phosphorus, for phasing has been possible for some systems, but hindered by the necessity to access longer X-ray wavelengths in order to make most use of the anomalous scattering contributions of these elements. Presented here are the results from a first successful experimental phasing study of a macromolecular crystal structure at a wavelength close to the sulfur K edge. This has been made possible by the in-vacuum setup and the long-wavelength optimised experimental setup at the I23 beamline at Diamond Light Source. In these early commissioning experiments only standard data collection and processing procedures have been applied, in particular no dedicated absorption correction has been used. Nevertheless the success of the experiment demonstrates that the capability to extract phase information can be even further improved once data collection protocols and data processing have been optimised.

## 1 Introduction

Structural biology as a field for understanding biological functions on an atomic level has expanded greatly during the sixty years of having high resolution models of macromolecules available. At the time of writing, the number of deposited structures in the protein data bank is larger than 120,000, of which most (>109,000) are based on data from X-ray crystallographic experiments [1].

The phase problem remains a major challenge in macromolecular X-ray crystallography. The intensities measured in a diffraction experiment only contain the information of the amplitudes of the complex structure factors, but not their phases. Without the phase information the Fourier transformation to calculate electron density maps in real space is not possible.

Most macromolecular crystal structures are nowadays solved by molecular replacement. With the growing database of macromolecular models, homologous molecules can be used for calculating initial phase estimations. The key tools for molecular replacement were developed in the 1960s [2] and the method has become predominant for macromolecular phasing today. However, the use of experimental phasing has continued to be needed for novel structures for which no homologous protein models are available or for validation purposes, when the risk of strong phase bias from a molecular replacement solution cannot be excluded.

In the first macromolecular structure determinations the crystallographic phase problem was solved by the multiple isomorphous replacement method (MIR). In MIR typically electron-rich elements bind to the macromolecule with the aim that changes to the measured diffraction intensities come from these introduced elements, while causing minimal disturbance to the remaining protein structure [3]. With multiple derivatives an unambiguous estimation of phases can be made. The concept of such difference measurements coming from a subset of atoms within each unit cell has remained as the main method for experimental phasing of macromolecules, but in slightly different forms.

Anomalous diffraction was early identified as a possible way of phasing macromolecules [4] and provides the benefit of not requiring multiple different isomorphous crystal structures from several heavy atom derivatives. It was first successfully applied for phasing the structure of the small protein crambin, using the anomalous diffraction from the sulfurs intrinsically present in the molecule [5]. This type of single wavelength experiment became known as the single-wavelength anomalous diffraction method (SAD). Also, during the 1980s, synchrotron light sources with the option to tune the X-ray wavelength enabled the use of anomalous diffraction from multiple wavelengths to solve the phase problem. This method uses the changes of the anomalous and dispersive contributions to the structure factors around an absorption edge from elements present in the crystal structure. This technique is called multiple wavelength anomalous dispersion (MAD) method [6], [7].

While MIR requires multiple heavy atom derivatives in isomorphous crystal forms, SAD and MAD can be performed on one crystal and can thereby avoid the necessity for isomorphous crystals. However, the presence of anomalous scatterers is needed. These scatterers can be introduced by soaking, co-crystallisation or biological incorporation of modified amino acids, such as seleno-methionine. An alternative to this is to make use of anomalous scattering from naturally occurring elements. For metalloproteins absorption edges lie typically within the wavelength range accessible at standard macromolecular crystallography beamlines (λ = 0.9 Å–2.5 Å). However, the edges of sulfur, which is present in the amino acids cysteine and methionine, and phosphorus, which forms part the RNA or DNA backbone, are at significantly longer wavelengths, at λ = 5.02 Å and 5.78 Å, respectively. Therefore sulfur and phosphorus based native SAD has remained inaccessible to many projects due to the very small anomalous signals present at shorter wavelengths. The anomalous signal increases approximately with the cube of the wavelength towards the sulfur and phosphorus K edges. Hence, long-wavelength native SAD experiments offer an opportunity to solve the phase problem directly from crystals without additional labelling. In recent years a combination of improved experimental setups on third generation synchrotrons has allowed successful native SAD studies from increasingly complicated structures using standard beamlines at wavelengths between 1.8 and 2.3 Å [8], [9], [10]. Only recently, new instruments have started to offer access to even longer wavelengths (2.7–3.3 Å) as at P13, PETRA III [11] and BL-1A, Photon Factory [12].

During the 1990s, proof of principle experiments were performed by H. Stuhrmann to utilise very long wavelengths for maximised anomalous differences. These experiments were performed in chambers with helium or air-gapped sample stages surrounded by vacuum, to minimise background scattering, and showed how some of the challenges with longer wavelength diffraction setups could be addressed [13], [14], [15], [16]. However, it was not possible to overcome all of them at this stage and further improvements on the beamline instrumentation were needed [17].

A dedicated macromolecular crystallography beamline, I23, for long-wavelength X-ray diffraction experiments, has been built at Diamond Light Source. I23 is designed to minimise background scattering and absorption by performing experiments in an in-vacuum end station, including the detector and sample environment. The semi-cylindrical Pilatus 12 M detector covers a large 2θ range of diffraction angels up to ±100°. Cooling of the crystals is realised by conductive links through the multi-axis goniometer in kappa geometry. Samples are transferred through a shuttle based air-lock system adapted from cryo-electron microscopy [18].

Here we present results from the ongoing commissioning work of this novel beamline at Diamond Light Source. A SAD experiment on a crystal from the protein thaumatin from *Thaumatococcus daniellii* was performed at a wavelength of 4.96 Å. While studies at similar wavelengths have previously been published [13], [14], [15], [16], we show the first successful phasing experiment at such a long wavelength, only 0.06 Å below the theoretical sulfur K edge.

## 2 Materials and methods

### 2.1 Crystallisation and sample handling

Thaumatin crystals were prepared as described in [18] changing the potassium/sodium tartrate concentration to 0.7 M. The crystal (approximately 110 × 60 × 60 μm^3^) used for data collection was harvested using a sample mount laser-cut from 10 μm thick glassy carbon Sigradur© (HTW, Thierhaupten Germany) and plunge-frozen in liquid nitrogen.

### 2.2 Data collection

A reference dataset was collected at a wavelength of 1.38 Å over a total range of 90°, followed by 400° of data at a wavelength of 4.96 Å. For both datasets diffraction images of 0.1° with 0.1 s exposure were recorded with the in-vacuum Pilatus 12 M detector in a continuous sweep. Datasets were collected with an unfocused beam of 300 × 300 μm^2^ in size, illuminating the whole crystal throughout the data collection. The flux of 1.6 × 10^11^ and 4.6 × 10^11^ photons/s, respectively, was determined by a diode positioned after the beam-defining slits. The temperature of the goniometer head was 43 K at the time of data collection, with an estimated temperature rise of 6 K across the thermal interface of the sample holder. Studies to accurately determine the sample temperature are currently being conducted.

Dose estimations were done with RADDOSE-3D [19] with a model of the crystal geometry generated in OpenSCAD [20]. The Bijvoet ratio of thaumatin for the different wavelengths was estimated as in [5].

### 2.3 Data processing and phasing

Data were processed with XDS [21]. No further attempts beyond the strict absorption correction model used in the CORRECT step of XDS were undertaken. The anomalous signal (|F(+) − F(−)|/σ) as a function of resolution was calculated with XSCALE [21]. For comparison of anomalous signals between the two different wavelength datasets, the first 90° of data for the λ = 4.96 Å dataset was processed and reported separately. For all further work, the complete 400° data range was used for the λ = 4.96 Å dataset. Substructure determination was performed with SHELXD [22], [23], using the λ = 4.96 Å dataset with 10,000 trials searching for 9 sites. Heavy atom sites were refined and used for phasing in SHARP [25] with density modification using DM and SOLOMON [26].

### 2.4 Initial model building

The density modified map together with the heavy atom sites were used for manually placing the cysteine and the methionine residues. Polyalanine chains were extended from these positions in Coot [27]. The model and phases were improved by iterating between phenix phase_and_build [24] and manual model building. Once roughly half the model had been accounted for, buccaneer [28] was able to trace the remaining residues with only minor registry and connectivity errors to manually correct for.

### 2.5 Refinement

Iterating between manual model building and refinement with phenix.refine [24], [29] started from the buccaneer model for the λ = 4.96 Å dataset and from PDB entry 4zg3 [18] for the λ = 1.38 Å dataset. MolProbity geometry validation [30] was used throughout the refinements. The selection of R_free_ reflections was imported from PDB entry 4zg3 for the λ = 1.38 Å dataset, while the λ = 4.96 Å dataset used a randomised selection of 10% of the reflections. B-factors were modelled isotropically per atom for the λ = 1.38 Å dataset, while the λ = 4.96 Å dataset was refined with one isotropic B-factor per amino acid and with secondary structure restraints. The model and structure factors of the λ = 4.96 Å dataset have been deposited in the Protein Data Bank under entry name 5TCL.

Distance comparison between the sulfur positions in the model and the SHELXD sites, used for phasing, was performed with phenix.emma [24].

### 2.6 Electron density map preparation

Phases from the density modified SHARP output were combined with the structure factor amplitudes from XDS using CAD [31] to generate anomalous difference maps with FFT [32]. This output was used for figure preparation with PyMOL [33]. Map correlation was calculated with get_cc_mtz_mtz in the phenix package [24], comparing maps calculated from the above mentioned merged file with the 2F_o_ − F_c_ map from the λ = 4.96 Å dataset phenix.refine output.

## 3 Results and discussion

**Fig. 1:**
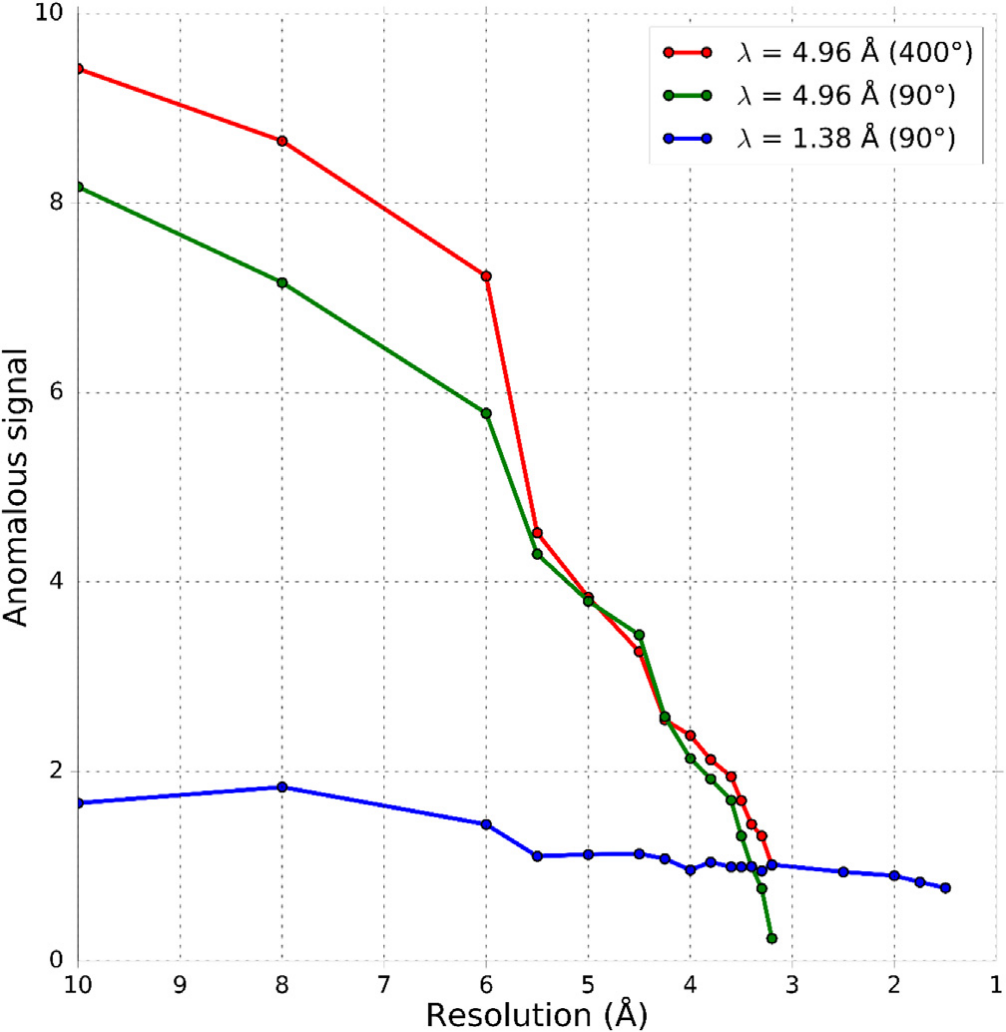
The anomalous signal, as reported by XSCALE, plotted as a function of resolution for the two datasets. Due to the λ = 4.96 Å dataset covering a larger rotation range than the λ = 1.38 Å dataset, data for both 90° and for 400° are shown. The lower anomalous signal for the 90° section of the λ = 4.96 Å dataset at higher resolution is an effect of the lowered completeness, due to the detector geometry.

The λ = 1.38 Å dataset first collected acts as a crystal quality indicator with the crystal diffracting to a resolution of 1.5 Å and a strong asymptotic *I*/*σ*(*I*) (ISa) of 65 (further statistics available in Table 1). The absorbed X-ray dose of 0.2 MGy was calculated by RADDOSE-3D for this first dataset. The λ = 4.96 Å dataset exposed the crystal to a 50 times higher dose of 11.4 MGy, which is still well within the Henderson limit of 20 MGy [34]. The maximum resolution at this very long wavelength is limited by the detector geometry to 3.2 Å, rather than the sample, and strong spots are seen to the edge of the corner detector panels. The detector geometry with an aspect ratio of 2:1 limits data completeness achievable with a single axis goniometer at highest resolution, depending on sample orientation and symmetry, which in this case leaves the outer shell with a low completeness of 75%. The ISa of 16 for the λ = 4.96 Å dataset also indicates a reduction of data quality compared to the λ = 1.38 Å dataset. This is also manifested in the R-factors in the same resolution shell (data not shown), which are significantly higher for the λ = 4.96 Å dataset. As the increase of the absorption cross section is around 45-fold when changing wavelength from λ = 1.38 Å to 4.96 Å, standard absorption correction protocols as used in XDS are no longer good enough to accurately correct for the absorption effects from the sample, sample mount and solvent. In fact the overall transmission through a path length of 60 μm in a protein crystal is less than 20%, so the overall data quality is surprisingly good. Nevertheless, a strong anomalous signal as predicted from the Bijvoet ratio of 8.8% for thaumatin, is present in the data despite the decreased data quality. This is highlighted in Fig. 1 with the anomalous signal as a function of resolution for the two different wavelengths.

**Table 1:** Data processing statistics from XDS and XSCALE with Friedel mates treated as separate reflections. The datasets were collected on the same crystal with the shorter wavelength being collected first. ^#^Calculated as in [5]. ^§^Accumulated dose as calculated by RADDOSE-3D for the whole crystal which fit inside the beam for all rotations. ^*^Wilson B calculated by phenix .table_one.

Data processing statistics	λ = 1.38 Å	λ = 4.96 Å
Photon energy (keV) [Thaumatin Bijvoet ratio^#^]	9.00 [1.0%]	2.50 [8.8%]
Rotational range (°)	90	400
Average dosage (MGy)^§^	0.2	11.4
Sulfurs/residues (disulfides)	17/207 (8)	
Space group and unit-cell (Å/°)	*P*4_1_2_1_2 58, 58, 151/90, 90, 90	
Resolution range (Å)	150.1–1.5 (1.6–1.5)	150.5–3.2 (3.4–3.2)
ISa	65	16
Wilson B (Å^2^)^∗^	14.8	38.8
Number of unique reflections	74,758 (12,881)	7 361 (986)
Multiplicity	3.2 (2.7)	9.5 (5.8)
Completeness (%)	96.6 (94.4)	91.7 (75.4)
R_merge_ (%)	7.8 (66.1)	7.5 (14.1)
R_meas_ (%)	9.2 (78.4)	8.0 (15.5)
SigAno	0.9 (0.8)	3.1 (1.1)
〈I/σ(I)〉	9.5 (1.2)	22.5 (9.4)
CC_1/2_ (%)	99.8 (52.7)	99.7 (98.6)

**Fig. 2:**
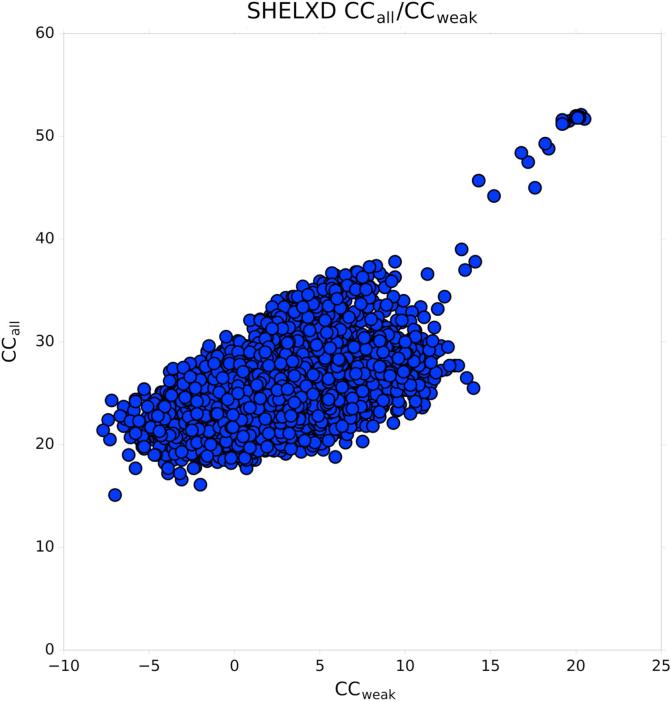
10,000 substructure solution attempts, searching for 9 sites, with SHELXD plotted with CC_weak_ vs CC_all_ for the λ = 4.96 Å dataset.

The sulfur substructure determination with SHELXD for the λ = 4.96 Å dataset was successful, as indicated by the separation of a second population with higher correlation coefficients in Fig. 2, with a success rate of 579 hits in 10,000 trials (5.8%). Due to the relatively low resolution limit of the long wavelength dataset of d_min_ = 3.2 Å disulfide bridges are not resolved and can be considered as super-sulfurs occupying single sites. Hence, 9 sites were found, as thaumatin contains 8 disulfide bridges and one methionine. A comparison of the substructure atom positions with the refined thaumatin sulfur positions shows that the substructure sites are positioned in between the two sulfur positions from the two cysteine residues forming the disulfide bridges (Table 2) and on the methionine*.*

**Table 2:** Distances in Ångström between the SHELXD solution used for phasing, to the closest sulfur atom in the refined model. For the disulfides the shortest distance within each disulfide to a site in the SHELXD solution is given. Distances calculated by phenix.emma.

Disulfide	Residue	Distance from refined sulfur positions to closest SHELXD site, per disulfide (Å)
1	Cys 9	0.8
	Cys 204	

2	Cys 56	0.4
	Cys 66	

3	Cys 71	1.0
	Cys 77	

4	Cys 121	0.9
	Cys 193	

5	Cys 126	0.6
	Cys 177	

6	Cys 134	1.0
	Cys 145	

7	Cys 149	0.7
	Cys 158	

8	Cys 159^∗^ (split conformations)	0.7
	Cys 164	

–	Met 112	0.1

**Fig. 3:**
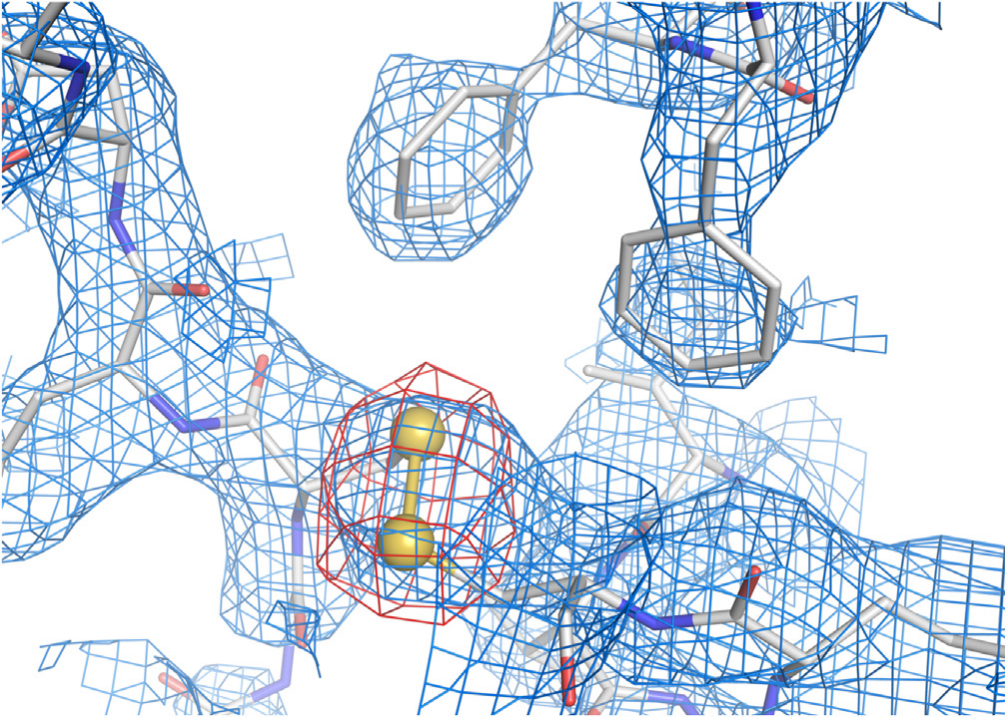
Experimentally phased map after density modification from SHARP in blue at 1.0 σ and an anomalous difference map at 5.0 σ. The refined thaumatin model is superposed (represented as sticks) to show the protein chain and the position of the sulfur atoms (yellow spheres) in the model. The SHARP map was carved 2.6 Å around the shown residues and the anomalous difference map was carved 3.0 Å around the sulfurs. (For interpretation of the references to color in this figure legend, the reader is referred to the web version of this article.)

**Fig. 4:**
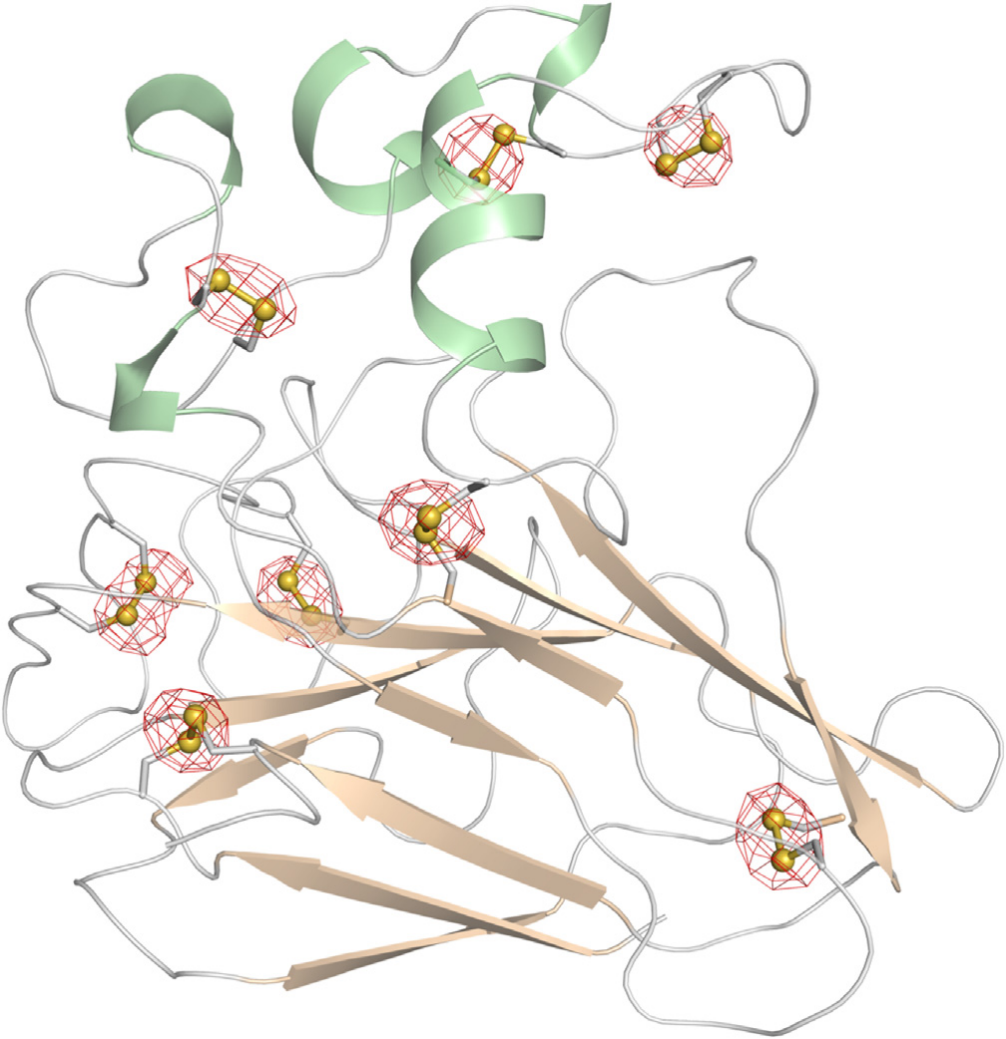
Experimentally phased anomalous difference map contoured at 5.0 σ shown with the refined model (cartoon representation, coloured by secondary structure) and the positions of the sulfur atoms as yellow spheres. The map was carved with a 2.5 Å cut-off around the sulfur positions. (For interpretation of the references to color in this figure legend, the reader is referred to the web version of this article.)

Phasing and density modification as performed with SHARP gave a map that carries several characteristic features of the protein backbone and aromatic residues as seen in Fig. 3. The anomalous difference Fourier map using these initial phases indicates the positions of all the sulfurs in thaumatin (Fig. 4). The experimental map connectivity and side chain features are generally good. The map correlation coefficient between the initial map based on the experimental phases and the final map after refinement is 0.725. This experimental electron density map at 3.2 Å resolution allowed to place a first protein model for manual building and subsequent model completion and refinement. In this first proof-of-principle experiment there has not been much optimisation of density modification parameters, or on the absorption correction during post-processing. Already without these optimisations the results show that datasets can be collected at such long wavelengths and be successfully used for substructure determination, phasing and refinement.

Model refinement against the λ = 1.38 Å dataset gave R-factors of 17/19% (*R/R_free_*) with mostly favourable geometry as seen in Table 3. For the λ = 4.96 Å dataset, no water molecules were built nor any split conformations, except for one tyrosine side chain otherwise causing strong positive difference map peaks. The reduced resolution for the long-wavelength dataset resulted in a lower number of reflections to refine against and only one B-factor per amino acid was refined. This resulted in refinement R-factors of 20/25% (*R/R_free_*).

**Table 3:** Refinement statistics from phenix.table_one. Friedel mates treated as separate reflections. High resolution shell values listed in parenthesis.

Refinement statistics	λ = 1.38 Å	λ = 4.96 Å
Resolution range (Å)	40.8–1.5 (1.6–1.5)	53.9–3.2 (3.3–3.2)
Reflections used in refinement	74,730 (7 412)	7 359 (571)
Reflections used for R-free	3 693 (397)	719 (57)
R-work	0.171 (0.336)	0.199 (0.292)
R-free	0.192 (0.364)	0.246 (0.322)
# atoms: total [protein/ligand]	1 875 (1 631/38)	1 575 (1 559/16)
RMS bonds (Å)/angles (°)	0.006/0.93	0.003/0.52
Ramachandran favoured/allowed/outlier (%)	99/1/0	97/3/0
Rotamer outliers (%)	0.6	0.0
Clashscore	1.5	2.0
Average B-factor (Å^2^)	20.4	29.1
Protein/ligand/solvent	18.9/27.0/30.5	29.1/25.2/–

## 4 Conclusions

This study shows that the I23 in-vacuum experimental setup enables crystallographic phasing of macromolecules at a wavelength close to the sulfur K edge. This first successful structure determination has been performed with standard crystallographic software packages, without specific adaptation to the unconventional experimental conditions, other than the detector geometry. This opens the door for harnessing the increased phasing power at these wavelengths by optimising data collection and data processing protocols. Dedicated absorption correction models will yield major improvements, while optimised data collection strategies, such as making use of multi axis goniometry, low-dose-high-multiplicity data collections and inverse beam datasets, will help to plan phasing experiments adequately. Altogether this warrants much improved data quality and novel experiments that previously were outside the reach of any other experimental setup.
